# CoCoNUT: an efficient system for the comparison and analysis of genomes

**DOI:** 10.1186/1471-2105-9-476

**Published:** 2008-11-12

**Authors:** Mohamed I Abouelhoda, Stefan Kurtz, Enno Ohlebusch

**Affiliations:** 1 Faculty of Engineering, Cairo University, Giza, Egypt; 2 Nile University, Giza, Egypt; 3 Center for Bioinformatics, University of Hamburg, Bundesstraße 43, 20146 Hamburg, Germany; 4 Faculty of Engineering and Computer Sciences, University of Ulm,89069 Ulm, Germany

## Abstract

**Background:**

Comparative genomics is the analysis and comparison of genomes from different species. This area of research is driven by the large number of sequenced genomes and heavily relies on efficient algorithms and software to perform pairwise and multiple genome comparisons.

**Results:**

Most of the software tools available are tailored for one specific task. In contrast, we have developed a novel system CoCoNUT (**C**omputational **C**omparative ge**N**omics **U**tility **T**oolkit) that allows solving several different tasks in a unified framework: (1) finding regions of high similarity among multiple genomic sequences and aligning them, (2) comparing two draft or multi-chromosomal genomes, (3) locating large segmental duplications in large genomic sequences, and (4) mapping cDNA/EST to genomic sequences.

**Conclusion:**

CoCoNUT is competitive with other software tools w.r.t. the quality of the results. The use of state of the art algorithms and data structures allows CoCoNUT to solve comparative genomics tasks more efficiently than previous tools. With the improved user interface (including an interactive visualization component), CoCoNUT provides a unified, versatile, and easy-to-use software tool for large scale studies in comparative genomics.

## Background

The size of genome sequence data has been rising at an exponential rate for the past decade or two, and will dramatically increase with new sequencing technologies becoming widely available. To analyze, annotate and compare these genome sequences, new algorithms and software for post-sequencing functional analysis are demanded by the scientific community.

Whole genome comparisons can be used as a first step toward solving genomic puzzles, such as determining coding regions, discovering regulatory signals, and deducing the mechanisms and history of genome evolution. Of importance to the genome annotation process, the genome comparison approach obviates the need for a priori knowledge of a protein sequence motif and provides a straightforward means for mapping information from the stored annotated genomes to the novel ones.

Sequence comparison in the context of comparative genomics is complicated by the fact that both local and global mutations of the DNA molecules occur during evolution. Local mutations (point mutations) consist of substitutions, insertions or deletions of single nucleotides, while global mutations (genome rearrangements) change the DNA molecules on a large scale. Global mutations include inversions, transpositions, and translocations as well as large-scale duplications, insertions, and deletions.

Thus, if the organisms under consideration are closely related (that is, if no or only a few genome rearrangements have occurred) or one compares regions which are suspected to be orthologous (regions in two or more genomes in which orthologous genes occur in the same order), then global alignments can, for example, be used for the prediction of genes and regulatory elements. This is because coding regions are relatively well preserved, while non-coding regions tend to show varying degrees of conservation. Genome comparisons of more closely related species may also help to determine the genetic basis for phenotype variation and may reveal species-specific regions (signatures) that can be targeted for identification.

For diverged genomic sequences, however, a global alignment strategy is likely predestined to failure for having to align non-colinear and unrelated regions.

In fact, the realm of comparative genomics is not limited to the comparison of two or multiple uni- or multi-chromosomal genomes. It also includes the comparison of two or multiple draft genomic sequences, the comparison of different assemblies, cDNA/EST mapping, and the comparison of two cDNA/EST libraries from different species. In all these tasks, the key problem is to identify regions of similarity among the sequences, and to align them.

To cope with the shear volume of data, most of the comparative genomics software-tools use an anchor-based method that is composed of three phases:

1. computation of fragments (segments in the sequences that are similar),

2. computation of highest-scoring chains of colinear non-overlapping fragments (these are the anchors that form the basis of the alignment), and

3. alignment of the regions between the anchors.

See [1,2] for reviews about the tools using this strategy for comparing whole genomic sequences, and see [3,4] and the references therein for the tools addressing the task of cDNA/EST mapping. All the tools employing this strategy implement the three phases, but the details depend on the task and are different among the tools. For example, some tools use exact algorithms, some use greedy algorithms, some use a graph based solution, and others use a geometric based solution.

### Comparative genome analysis on a large scale

A tool for the systematic comparative study of sequences as large as vertebrate or plant genomes must satisfy the following criteria.

#### Versatility

To be useful for molecular biologists, such a tool should be able to deal with versatile tasks. The CoCoNUT system supports the following genome comparison tasks:

• Computation of a multiple alignment of closely related (i.e., similar) sequences.

• Computation of regions of high similarity among multiple genomic sequences.

• Comparison of two draft or multi-chromosomal genomes. (This task is similar to the comparison of two cDNA/EST libraries).

• Identification of segmental duplications in whole genomic sequences.

• cDNA/EST mapping.

To the best of our knowledge, there is no other software-tool which covers so many tasks. CoCoNUT is freely available for non-commercial purposes.

#### Compositionality and usability

A complex system supporting the manifold tasks of genome analysis usually consists of several advanced programs. Thus it must provide simple interfaces to enable the composition of these programs. CoCoNUT uses variations of the above-mentioned anchor-based strategy to support genome comparison tasks. The three phases (1) computation of fragments, (2) chaining of fragments, and (3) post-processing of chains are clearly separated. Thus, it is possible to exchange a program performing one of the phases without affecting the whole system. Moreover, it is possible to stop the computation at any phase, and store the intermediate results for later use.

#### Efficiency

To analyze complete genomes of up to several billion base pairs, the space and time used by the algorithms must scale "almost" linearly with the sequence length and the output size. CoCoNUT is based on the anchor based strategy mentioned before and its algorithms meet this requirement. Our implementation of the crucial first phase (computations of fragments) is linear, see [5] for more details. The second phase uses techniques from computational geometry to chain the fragments. This approach is "almost" linear in the number of fragments, which is a considerable advantage over the straightforward graph based approach (which requires quadratic running time). For more details about our chaining algorithms, see [4,6-8].

Experimental results show that CoCoNUT is able to efficiently handle large sequence sets. For example, four bacterial genomes are processed (i.e., finding regions of high similarity and aligning them) in a few minutes. CoCoNUT can process three large mammalian X-chromosomes in about one hour on a standard workstation. However, for the largest vertebrate chromosomes, a server class machine (of say 16 or 32 GB of RAM) is probably required. Comparing a set of complete mammalian genomes in one run would require even more RAM, and therefore we recommend to perform the comparison on the chromosome level. However, a general statement about the upper limit of the number and size of the sequences which can be processed is difficult, because the resource requirement very much depends on the similarity of the genomes. The more similar, the more matches are to be computed and the more resources are required.

#### Interactive Visualization

The large amount of data delivered by comparative genomics requires a visualization. CoCoNUT comes with an interactive visualization tool called VisCHAINER. This displays dot plots of the comparison results (with zoom and select capabilities). In contrast to established dot-plot tools like DOTTER [9] or Gepard [10], VisCHAINER can automatically display dot plots of multiple genome comparisons. That is, all two-dimensional projections of the common regions among multiple genomes are plotted. Moreover, VisCHAINER has functionalities specific to the anchor-based strategy.

### Related work

#### Whole genome comparison

In [2], Treangen and Messeguer presented a classification of genome comparison tools. In this classification, CoCoNUT falls into the category of large-scale multiple genome comparison tools. Some of these (ABA [11], Mulan [12], TBA [13], Mauve [14], and M-GCAT [2]) can deal with genome rearrangements. In what follows, we briefly compare our system with these software-tools except for Mulan, because Mulan is a network server based on TBA. ABA and TBA employ a progressive alignment strategy, i.e., they construct local alignments from pairwise comparisons, possibly following a "guide" tree. Both tools use BLASTZ [15] to identify hits (small regions of similarity) between pairs of genomes, and then they combine these hits into larger alignment blocks. Therefore, these tools can detect similarities that must not necessarily be present in all of the genomes under consideration. This is an advantage over Mauve, M-GCAT, and CoCoNUT. On the other hand, both tools suffer from large running times even for short sequences; see [11,13] and [[2], page 2].

Mauve and M-GCAT use maximal unique matches as fragments. By definition, these matches occur only once in each genome (or chromosome). As a consequence, for genomes containing large-scale duplications (e.g., plants genomes), the number of fragments may be very small and thus no reasonable alignment can be produced. In fact, this shortcoming was already mentioned in [16] and [2].

#### Identification of large genomic duplications

There are many software tools for locating repeated segments in large genomic sequences; see [17] for a review. CoCoNUT is different from other tools because it can efficiently locate large genomic duplications (such as di- and tetraploidization). These are difficult to detect as they (1) are very long, (2) may be interrupted by large gaps (due to deletions or insertions of other repeat types), and (3) might have undergone rearrangement events. As an example, we show how CoCoNUT can locate the genome duplications in chromosome I of *A. thaliana*.

#### cDNA/EST mapping

Standard dynamic programming algorithms cannot be used for high throughput mapping of cDNA sequences because they have a quadratic running time. Hence, heuristic algorithms have been developed for this task. All of them either use a seed-and-extend strategy or a chaining strategy.

Tools applying the seed-and-extend strategy include, among others, BLAT [18] and MGAlign [19]. These tools differ in the type of seeds they use and in the way the seeds are computed. Tools using the chaining based strategy include, among others, GenomeThreader [20], GMAP [21] and the program by Shibuya and Kurochkin [3].

The work of [3] is worth mentioning because it uses suffix trees for the computation of exact matches and introduces a geometric-based chaining algorithm. In [4], the algorithm of [3] was further refined by using enhanced suffix arrays instead of suffix trees, by using maximal exact matches instead of maximal unique matches, and by using a chaining algorithm that is less complicated and more suitable for cDNA mapping. Its sensitivity/specificity was compared to the program BLAT (the most popular program for cDNA mapping applying the *k*-mer based seed-and-extend strategy). It was shown in [4] that the chaining strategy is more specific than the seed-and-extend strategy, while achieving the same level of sensitivity. Moreover, the algorithm obviates the need for masking the genomes, while unmasked sequences can often not be processed by *k*-mer based seed-and-extend strategies, as the number of *k*-mers is too large. Seed-and-extend strategies based on maximal exact matches (e.g., as implemented in [22]) may be able to process unmasked, sequences, but for cDNA mapping they are less specific than the chaining approach, see above. In CoCoNUT, the algorithms and software prototypes presented in [4] were rewritten and extended by additional splice site detection methods.

## Implementation

### Computing the fragments

For *i*, 1 ≤ *i *≤ *k*, let *S*_*i *_= *S*_*i*_[1..*n*_*i*_] denote a string of length *n*_*i *_= |*S*_*i*_|. In our applications, *S*_*i *_is a long DNA sequence (e.g., a complete chromosome). Furthermore, *S*_*i*_[*l*_*i*_... *h*_*i*_] is the substring of *S*_*i *_starting at position *l*_*i *_and ending at position *h*_*i*_. A fragment consists of substrings *S*_1_[*l*_1_... *h*_1_], *S*_2_[*l*_2_... *h*_2_],..., *S*_*k*_[*l*_*k*_... *h*_*k*_] that are "similar". If *S*_1_[*l*_1_... *h*_1_] = *S*_2_[*l*_2_... *h*_2_] = ... = *S*_*k*_[*l*_*k*_... *h*_*k*_] (i.e., the substrings are identical), then such a fragment is called *exact fragment *or *multiple exact match*. A multiple exact match is called *left maximal*, if *S*_*i*_[*l*_*i *_- 1] ≠ *S*_*j*_[*l*_*j *_- 1] for some *i *≠ *j*, and it is called *right maximal *if *S*_*i*_[*h*_*i *_+ 1] ≠ *S*_*j*_[*h*_*j *_+ 1] for some *i *≠ *j*. A *multiple maximal exact match *(*multiMEM *for short) is left and right maximal. In other words, the constituent substrings cannot be simultaneously extended to the left and to the right.

A *multiMEM *is called *rare *if the constituent substrings *S*_*i*_[*l*_*i*_... *h*_*i*_] appear at most *r *times in *S*_*i*_, where 1 ≤ *i *≤ *k *and *r *is a natural number specified by the user. We call the value *r *the *rareness *value. A *multiMEM *is called *unique *if *r *= 1. In this case, we speak of a *multiple maximal unique match *or *multiMUM *for short. Note that the maximal unique matches used in the program MUMmer can be viewed as *multiMEMs *with rareness value *r *= 1 for *k *= 2 sequences.

If character mismatches, deletions, or insertions are allowed in the constituent substrings of a fragment, then we speak of a *non-exact fragment*. The programs DIALIGN [23] and LAGAN [24] compute fragments with substitutions, and the program BLASTZ [15] (used in PipMaker [25]) computes fragments with substitutions, insertions, and deletions.

Our system can use any kind of fragments, provided that they are output in the CoCoNUT format. For our experiments, we use (rare) *multiMEMs *because these are easier and faster to compute than non-exact matches. Using spaced seeds [26] for pairwise comparisons would also be reasonable. Note that *multiMEMs *of minimum length *k *achieve the same level of sensitivity as approximate matches computed by extending seeds of length *k*. Moreover, the number of *multiMEMs *is much smaller than the number of *k*-mers, which results in faster processing and better specificity.

**Figure 1:**
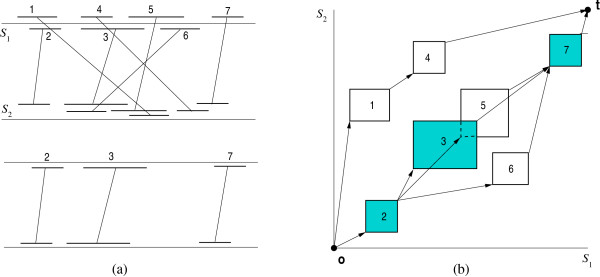
**Fragment representation and global chaining**. The fragments in (a) can be represented, as shown in (b), by hyper-rectangles in a *k*-dimensional space, where *k *is the number of genomes, and each axis corresponds to one genome. In this example, the optimal global chain of colinear non-overlapping fragments consists of the fragments 2, 3, and 7.

Geometrically, a fragment *f *of *k *genomes can be represented by a hyper-rectangle in ${\mathbb{N}}_{\geq 0}^{k}$ with the two extreme corner points *beg*(*f*) and *end*(*f*). *beg*(*f*) is the *k*-tuple (*l*_1_, *l*_2_,..., *l*_*k*_), where *l*_1_,..., *l*_*k *_are the start positions of the fragments in *S*_1_,..., *S*_*k*_. *end*(*f*) is the *k*-tuple (*h*_1_, *h*_2_,..., *h*_*k*_), where *h*_1_,..., *h*_*k *_are the end positions of the fragments in *S*_1_,..., *S*_*k*_, respectively; see Figure 1. With every fragment *f*, we associate a positive weight *f. weight *∈ ℝ. This weight can, for example, be the length of the fragment (in case of exact fragments) or its statistical significance. In our system, in the default case, we use the fragment length as weight.

### Chaining the fragments

We define a binary relation ≪ on the set of fragments by *f *≪ *f' *if and only if *end*(*f*).*x*_*i *_<*beg*(*f'*).*x*_*i *_for all *i*, 1 ≤ *i *≤ *k*. If *f *≪ *f'*, then *f precedes f'*. Two fragments in a chain are *colinear *if the order of their respective segments is the same in all genomes. In the pictorial representation of Figure 1(a), two fragments are colinear if the lines connecting their segments are non-crossing (e.g., the fragments 1 and 4 are colinear, while 1 and 2 are not).

A *chain *of colinear non-overlapping fragments (or chain for short) is a sequence of fragments *f*_1_, *f*_2_,..., *f*_ℓ _such that *f*_*i *_≪ *f*_*i*+1 _for all 1 ≤ *i *< ℓ. The *score *of a chain ⟨*C *= *f*_1_, *f*_2_,..., *f*_ℓ_⟩ is

$$score(C)=\sum_{i=1}^{\ell }f_{i}.weight-\sum_{i=1}^{\ell -1}g(f_{i+1},f_{i})$$

where *g*(*f*_*i*+1_, *f*_*i*_) is the cost of connecting fragment *f*_*i *_to *f*_*i*+1 _in the chain. We will call this cost *gap cost*. The gap cost implemented in the current version of CoCoNUT is defined as follows. For two fragments *f *≪ *f'*,

$$g(f^{\prime },f)=\sum_{i=1}^{k}\left|beg(f^{\prime }).x_{i}-end(f).x_{i}\right|$$

**Figure 2:**
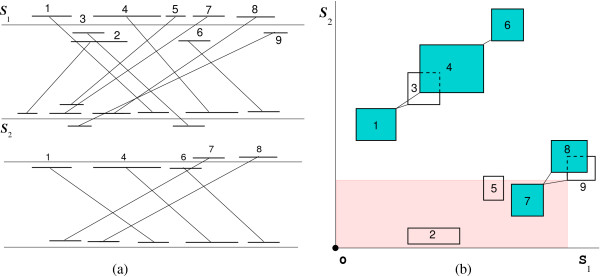
**Local chaining**. Computation of an optimal local chain of colinear non-overlapping fragments. The optimal local chain is composed of the fragments 1, 4, and 6. When the start point of fragment 9 is scanned, a range maximum query searches for a fragment of highest score occurring in the shaded region.

Given *n *weighted fragments from two or more genomes, the following problems can be defined:

• The *global chaining problem *is to determine a chain of maximum score starting at the origin 0 = (0,..., 0) and ending at the terminus point t = (*n*_1 _+ 1,..., *n*_*k *_+ 1). Such a chain will be called *optimal global chain*. Figure 1 shows a set of fragments and an optimal global chain.

• The *local chaining problem *is to determine a chain of maximum score ≥ 0. Such a chain will be called *optimal local chain*. It is not necessary that this chain starts at the origin or ends at the terminus. Figure 2 shows a set of fragments and an optimal local chain.

• Given a threshold *T*, the *all significant local chains problem *is to determine all chains of score ≥ *T*. Obviously, the all significant local chains problem is a generalization of the local chaining problem.

In a solution to the all significant local chains problem, some chains can share one or more fragments, composing a cluster of fragments. In the example of Figure 2, the local chains ⟨1, 3, 6⟩ and ⟨1, 4, 6⟩ share the fragments 1 and 6, yielding the cluster ⟨1, {3, 4}, 6⟩. The cluster ⟨7, {8, 9}⟩ represents two local chains ⟨7, 8⟩ and ⟨7, 9⟩. To reduce the output size, we report the clusters and from each cluster we report a local chain of highest score as a representative chain of this cluster. This representative chain is a significant local chain. In the example, the representative chains are ⟨1, 4, 6⟩ and ⟨7, 8⟩.

Our chaining algorithm is not heuristic, i.e., it computes an optimal chain w.r.t. the given constraints. It is based on the line-sweep paradigm and uses *range maximum queries *(*RMQ*) with activation. During the line sweep procedure, the fragments are scanned w.r.t. their order in one of the genomes. If an end point of a fragment is scanned, then it is activated. If a start point of a fragment is scanned, then we connect it to an activated fragment of highest score occurring in the rectangular region bounded by the start point of the fragment and the origin. This highest-scoring fragment is found by an *RMQ*, see Figure 2(b). For more details about our chaining algorithms; see [6,7,27]. In practice, variations of the basic algorithms or certain pre-processing steps are required. Because these variations are specific to each application, we handle them in detail in the respective sections.

### The data flow in CoCoNUT

**Figure 3:**
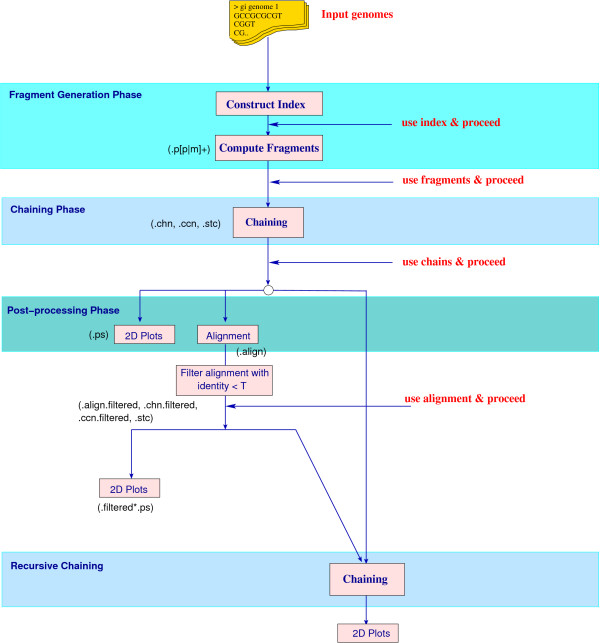
**CoCoNUT block diagram**. The data-flow in CoCoNUT for the task of comparing multiple genomes. The user can repeat the comparison starting in any of the four phases (marked as *use index*, *use fragments*, *use chains*, and *use alignment*) and proceed further in the comparison. The extensions of the files produced in each step are shown in brackets; see also Table 1.

Figure 3 summarizes the data flow and typical usage of CoCoNUT.

The input to the system is a set of genomic sequences. For genome analysis, all chromosomes are input. Usually, each chromosome is given as a single FASTA file and one compares a combination of chromosomes at a time. (However, CoCoNUT can also compare two multi-chromosomal or draft genomes in a single run; this will be explained in the following section.) Inversions can be taken into account by considering the backward strands of some chromosomes and the forward strands of the other chromosomes. In CoCoNUT, all combinations of orientations are considered by default, but the user has the option to restrict the comparison to the forward strands only. For repeat analysis, the input is a single genomic sequence in single or multiple FASTA files. For cDNA mapping, the user submits one genomic sequence and cDNA sequences in a multiple FASTA file.

Each comparison consists of a fragment generation phase and a chaining phase. The fragments are usually generated by the program ramaco [5], which computes rare *multiMEMs *using an enhanced suffix array of one of the chromosomes. Alternatively, if one does not expect too many repeats in the considered sequences (and therefore no explosion in the number of *multiMEMs*), it may not be necessary to specify a rareness parameter. In such a case, one can use the program multimat [28] to compute the *multiMEMs*. While ramaco can also compute *multiMEMs *(without rareness parameters), multimat does this more efficiently at the expense of a larger index size (as it requires to build an enhanced suffix array of all chromosomes.) The program CHAINER carries out the chaining phase and delivers all significant local chains, where each chain corresponds to a region of similarity.

Upon completion of these two phases, CoCoNUT provides the functionality to

• visualize the resulting chains in 2D plots, or

• compute an alignment on the nucleotide level for each chain (by the strategy described in the next section) and filter out chains with low sequence identity, or

• compute and visualize regions of high similarity, or

• perform a second chaining step with the chains of the first chaining step as new fragments.

To repeat parts of the comparison with different parameters, the user can re-start the comparison at four points: (1) after the index generation, (2) after the fragment generation, (3) after the first chaining step, and (4) after the alignment. For example, if the user has already computed the fragments and chains, then he/she could run the alignment program later, based on the stored fragments and chains. He/she could also repeat the chaining step with the stored fragments, but with different parameters.

## Results and discussion

### Finding regions of high similarity

The first two phases of CoCoNUT are (1) the computation of fragments (*multiMEMs*) and (2) the computation of all significant local chains. These chains correspond to regions of high similarity, but the reader should keep in mind that the regions depend on the parameters with which the program was called. This behavior bears resemblance to the widely used program BLAST [29] for comparing DNA or protein sequences. A BLAST search enables a researcher to compare a query sequence with a database of sequences, and identify sequences in the database that are similar to the query sequence. The sequences delivered by BLAST depend on the parameters with which the program was called, and the parameter choice is very important. The following scenario shows a typical usage of BLAST. Following the sequencing of a DNA segment of functional importance in a certain species, a scientist will typically perform a BLAST search against genomes of related species. It is then a research hypothesis that the sequences identified by the search are in fact homologous (in the phylogenetic sense) to the query sequence. However, because sequence similarity may arise from different ancestors (e.g., short sequences may be similar by chance or sequences may be similar because both were selected to bind to a particular protein, such as a transcription factor) this working hypothesis must be corroborated. The same is true for CoCoNUT. The regions of high similarity identified by CoCoNUT may or may not be homologous, and an alignment of these may or may not be meaningful.

Other authors use the terms *synteny *or *syntenic regions *instead of *regions of high similarity*. In genetics, *synteny *describes the physical co-localization of genetic loci on the same chromosome within an individual or species, while *shared synteny *describes preserved co-localization of genes on chromosomes of related species. The term *shared synteny *is sometimes also used to describe preservation of the precise order of genes on a chromosome passed down from a common ancestor, but many geneticists reject this use of the term. Passarge et al. [30] wrote: "We believe molecular biologists ought to respect the original definition of synteny and its etymological derivation, especially as this term is still needed to refer to genes located on the same chromosome. We recognize the need to refer to gene loci of common ancestry. Correct terms exist: 'paralogous' for genes that arose from a common ancestor gene within one species and 'orthologous' for the same gene in different species." However, in our context, the term *orthologous regions *cannot be used either, simply because we cannot generally infer orthology from sequence similarity alone (nevertheless, shared synteny in the gene order sense is one of the most reliable criteria for establishing the orthology of genomic regions in different species). Because there is no "right word" yet, we will use the term *regions of high similarity*, although we feel that this term does not have the right connotation (especially if one uses a second chaining step, see below).

In contrast to global alignment tools (e.g., MGA [31]), which assume global similarity, CoCoNUT can cope with genome rearrangements. It uses the three step approach depicted in Figure 3. The user can specify several parameters in the CoCoNUT system and a reasonable parameter choice is very important.

In the fragment generation phase, the parameter "minimum fragment length" can be set by the user, but it is usually a good idea to first use the default parameter, which is estimated based on the count statistics; see [32]. Furthermore, the user can specify the rareness value of a fragment. The rareness parameter depends on the number of "important to see" repeated segments in the genomes, an information that cannot be determined automatically. Therefore, the user has to test different rareness values. In our experiments, we found that 5 is a reasonable rareness value to start with.

Only fragments (*multiMEMs*) whose lengths exceed the minimum fragment length are generated. On the one hand, if the minimum fragment length is too small or the rareness value is too large, a large number of fragments is generated. On the other hand, if the minimum fragment length is too large or the rareness value is too small, too few fragments for a meaningful comparison may be generated.

In the chaining phase, CHAINER solves the *all significant local chains problem*. In addition, the user can specify an upper bound on the gap length between fragments in a chain. That is, two fragments can only be connected in a chain if the number of characters separating them does not exceed this user-defined maximum gap-length, which is identical for all sequences. This option prevents unrelated fragments from extending a chain. The user can also filter out chains based on their length or their score. (This filtration can also be carried out using the visualization tool VisCHAINER.)

The fragments of a local chain represent anchors that form the basis of the local alignment. Only the regions between them must be aligned on the nucleotide level. If one compares just two genomes, the regions between the anchors are aligned by a global alignment algorithm based on standard dynamic programming. For more than two genomes, the program CLUSTALW [33] is used to align these regions. This strategy is also used in the multiple global alignment tool MGA [31], albeit for a single global chain.

For closely related genomes, it is recommended to increase the minimum fragment length. This usually does not affect the sensitivity of the procedure. Moreover, a single chaining step is usually enough to identify regions of high similarity.

For distantly related genomes, the minimum fragment length needs to be reduced to increase the sensitivity of the comparison. This, however, has the effect that many fragments appear by chance. To identify regions of high similarity in the "noisy" fragment set, it is important to use a double-chaining strategy. In the first chaining phase, one computes chains of *multiMEMs *with a stringent gap length. In the second chaining phase, the chains resulting from the first chaining step are considered as new fragments. Moreover, the gap length is increased. In this way, it is possible to remove the noise without missing relevant fragments.

We exemplify these two strategies by comparing three related bacterial genomes and three distantly related mammalian chromosomes. The experiments were carried out on a Sun Sparc V processor with 1015 MHz and 6GB RAM.

### Comparing closely related bacterial genomes

**Figure 4:**
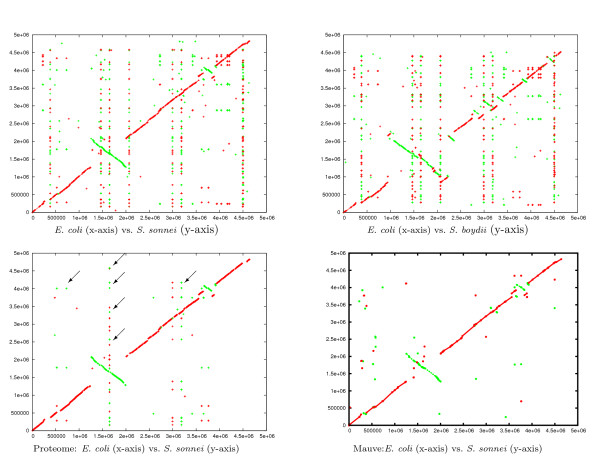
**Comparison of three bacterial genomes**. A comparison of the three bacterial genomes of *E. coli*, *S. sonnei*, and *S. boydii*. We show the projections *E. coli vs. S. sonnei *(upper left) and *E. coli vs. S. boydii *(upper right). (The third projection *S. sonnei vs. S. boydii *is not shown). The plot on the lower-left is the projection *E. coli *vs. *S. sonnei*, where each point represents a protein that is encoded in all three genomes. (Other projections are not shown.). The arrows point to some proteins repeated in the genomes (vertically aligned hits correspond to repetitions in *S. sonnei *and the horizontally aligned ones correspond to repetitions in *E. coli*.). The fourth plot is the projection *E. coli vs. S. sonnei *plotted according to the alignment computed by the program Mauve. Red lines correspond to similar regions (or protein hits) between the forward strands of the genomes on the x-and y-axis, while green lines correspond to similar regions (or protein hits) between the forward strand of the genomes on the x-axis and the backward strand on the y-axis.

We compared the three bacterial genomes *E. coli, S. sonnei*, and *S. boydii *(accession numbers are NC_000913, NC_007384, and NC_007613, respectively). As a reference, we first compared the proteomes of the three genomes and obtained the best hit of all proteins encoded in three genomes. This comparison was performed using the *Comprehensive Microbial Resource *web-based comparison tools [34]. We used the option that reports the best hits for each protein. Figure 4 (lower left) shows the projection *E. coli vs. E. sonnei *in which the hits that appear on a vertical or horizontal line correspond to repeated segments in *E. coli *or *E. sonnei *encoding the same protein. (By searching the non-redundant protein database using BLAST, we found that the repeats are insertion elements.)

We used CoCoNUT to compare the three genomes on the DNA level. The minimum length of the fragments was between 15 to 18 and the rareness value was between 5 and 20. (The default values for minimum length and rareness are 18 and 5, respectively.) In the chaining step, the maximum gap length was set to 1000 bp. For minimum length 15 and rareness value 20, we obtained the best results w.r.t. the reference comparison on the protein level. All chains of length less than 500 bp were filtered out. As can be seen in Figure 4, the regions containing orthologs are covered by the local chains. The remaining repeated segments visible in the DNA plot, but not in the protein plot, are insertion elements that do not encode a protein.

In this comparison, there was no need for a second chaining step because regions of high similarity could easily be identified. All alignments derived from the chains show an identity of more than 70%. The whole experiment, including the computation of the multiple alignment, took a few minutes.

We applied the program Mauve to the same three bacterial genomes. Mauve uses fragments of the type *multiMUMs*, and as shown in Figure 4, is not able to identify repeated segments.

### Comparing distantly related mammalian chromosomes

The X-chromosomes of human, mouse, and rat were compared by CoCoNUT. We used masked and unmasked sequences of the latest assemblies of the three genomes. We used the human genome version 18 NCBI build 36, the mouse genome version 9 NCBI build 37, and the rat X-chromosome version 4 RGSC v3.4 from the UCSC genome browser. The accession numbers of the X-chromosomes of human, mouse, and rat are NC_000023 and NC_000086, and NC_005120, respectively.

**Figure 5:**
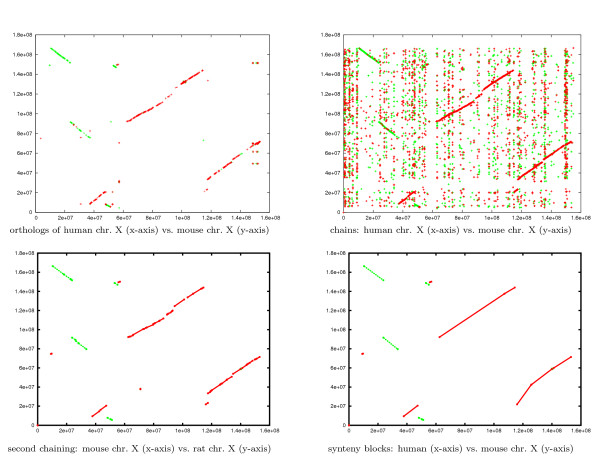
**The similar regions of three mammalian chromosomes**. The results of comparing the three unmasked mammalian X chromosomes, projected w.r.t. the human and mouse chromosomes. (Other projections are not shown.) Red lines correspond to chains between the forward strands of the X-chromosomes on the x- and y-axis. Green lines correspond to chains between the forward strand of the X-chromosome on the x-axis and the backward strand of the X-chromosome on the y-axis, i.e., they correspond to inversions. The plot on the upper left shows projection of the orthologous proteins w.r.t. the human and mouse chromosomes. (Different colors refer to different orientations.) The plot on the upper right shows projections of the resulting three dimensional chains w.r.t. the human and mouse chromosomes. The plot on the lower left shows the results of the double-chaining strategy. The lower right plot shows the synteny blocks computed by CoCoNUT.

As a reference, we used the BioMart system [35,36] to retrieve all orthologous proteins among the three X-chromosomes. The human X-chromosome was taken as a reference. Figure 5 (upper left) shows the plot for the human vs. mouse X-chromosome. (Projections w.r.t. other pairwise genomes are not shown.) Each point in this plot corresponds to a protein shared in all X-chromosomes with identity larger than 25%.

We also compared our results with Bourque et al. [37], who identified *synteny blocks *and used them to compute genome rearrangement scenarios. (A synteny block in the Bourque et al. paper is composed of non-repeated colinear regions of high similarity. As discussed above, some readers may reject the use of the term *synteny block*, but we will stick to the original terminology used by Bourque et al. [37].) The synteny blocks were identified by first combining the results of all pairwise genome comparisons, and then by verifying these using all pairwise proteome comparisons.

We ran CoCoNUT using different parameters, starting with the default values. The parameters that produced good results were as follows: The minimum fragment length was 20 and the rareness value was 10. Furthermore, the gap length between two fragments in a chain was set to 600 bp and chains with length less than 80 bp were filtered out. Figure 5 (upper right) shows the projections of the resulting chains w.r.t. the human and mouse chromosomes. (Other projections are not shown.)

Although the results show that the regions containing the orthologous proteins are covered by CoCoNUT local alignments, it is difficult to automatically identify large regions of high similarity by visual inspection. Therefore, we performed a second chaining step. In this step, the gap length between two fragments was 500 Kbp, and chains with length less than 300 Kbp were filtered out. The resulting chains were considered to be the regions of high similarity. The parameters were chosen to mimic the strategy of [37]. Figure 5 (lower left) depicts the results of the second chaining step w.r.t. the human and mouse chromosomes. (Other projections are not shown.) Table 1 in Additional file 1 lists the exact coordinates of the regions of high similarity.

**Table 1:** File extensions for CoCoNUT output and their semantics

extension	file contents
.chn	chains
.ccn	chain boundaries without fragments
.stc	statistics about the fragments and the chains
.align	alignment of the chains on a character level
.filtered	filtered chains
.ps	2D plots of the results in PostScript format

CoCoNUT can optionally filter out repetitions and coalesce the regions of high similarity into synteny blocks. The corresponding output is shown in Figure 5 (lower right); see Table 2 in Additional file 1 for the exact coordinates. A detailed analysis of the genome comparisons of the three X-chromosomes reveals that our results are very similar to the results presented in [37] (the boundaries of the synteny blocks differ only slightly), except for two segments that are not in the same orientation. We attribute this difference to the fact that the genome assembly we used is a more recent one compared to [37]. In the new assembly, the X-chromosome sizes were modified and the orientation of two large segments in the mouse X-chromosome was corrected. (This can be verified by comparisons on the protein level; see Figure 5). The reason why we preferred to use the new assemblies is to stimulate a follow-up study for re-estimating the rearrangements scenario using the new synteny blocks.

For masked X-chromosomes, the fragment generation and the chaining step took about 21 minutes, and the alignment step took about 40 minutes. For unmasked X-chromosomes, the fragment generation and the chaining step took about 95 minutes, and the alignment step took 47 minutes. The time for the second chaining step is a few seconds.

We can conclude from this example that the use of rare *multiMEMs *permits CoCoNUT, unlike other tools such as BLASTZ, to work with complete unmasked sequences. Although the computation of rare *multiMEMs *takes most of the time of the genome comparison, it is still much faster than masking and processing the sequence using other tools.

### Comparing two draft/multi-chromosomal genomes

In contrast to a finished genome, a draft genome consists of contigs of unknown order and orientation (a contig is a contiguous stretch of the genome). Unlike any other software tool, CoCoNUT can compare *two *draft or multi-chromosomal genomes in a single run, i.e., without the explicit comparison of all pairs of contigs/chromosomes. Although there is no theoretical advantage in running the experiment at once, it is still an attractive feature, as it does not require to artificially split up a collection of sequences. Less important, but worth to mention, is that it is slightly faster because the comparison runs at once in main memory, and no repeated access to the external memory for each pairwise comparison is required. The disadvantage of this feature is the increased space consumption, due to the fact that the complete sequence set is stored as an enhanced suffix array.

**Figure 6:**
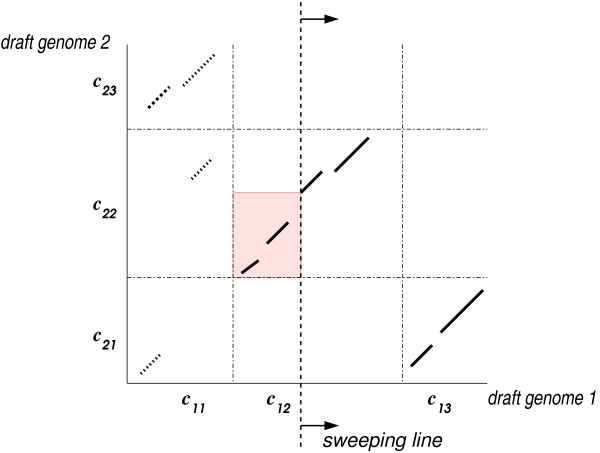
**Chaining for draft/multi-chromosomal genomes**. The contigs *c*_11_, *c*_12 _and *c*_13 _of the first draft genome are compared to the contigs *c*_21_, *c*_22 _and *c*_23 _of the second draft genome. The dash-dotted lines represent the borders between the contigs. The range maximum query is restricted to the colored region, because the fragments in the contigs *c*_12 _× *c*_22 _are chained.

We proceed as depicted in Figure 3, but now the boundaries between the contigs/chromosomes are taken into account. More precisely, all contigs/chromosomes of each draft/multi-chromosomal genome are concatenated, but a unique separator symbol is inserted between consecutive contigs/chromosomes to represent their border. The fragments are then generated w.r.t. the concatenated sequences. This allows us to use the same line-sweep algorithm as in the basic chaining algorithm, but we have to make sure that the chains do not cross the borders between contigs/chromosomes. This can be done by restricting a range maximum query to the fragments lying in the same contig/chromosome; see Figure 6. Regions of high similarity and synteny blocks are computed as described in the previous sections, but the contig/chromosome boundaries are taken into account.

**Figure 7:**
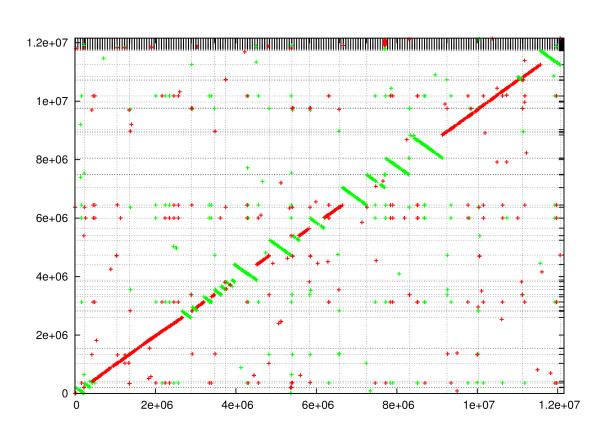
**Comparing a draft genome to a finished genome**. Comparison of the finished multi-chromosomal budding yeast genome (x-axis) and the draft genome of *S. paradoxus *(y-axis). The vertical lines correspond to the boundaries between the chromosomes. The horizontal lines corresponds to the boundaries between the scaffolds (333 scaffolds).

To exemplify this, we compared the finished multi-chromosomal genome of *S. cerevisiae *and the draft genome of *S. paradoxus*. (The *S. cerevisiae *genome consists of 16 chromosomes and the mitochondrial genome. Accession numbers are from NC_001133 to NC_001148 and NC_001224. The *S. paradoxus *genome consists of 333 scaffolds from 832 contigs assembled in [38,39]. The contigs are deposited in Genbank with accession numbers from AABY01000001 to AABY01000832). Figure 7 shows the result of this comparison. The plot shows a high similarity between the two genomes. (In the comparison, the minimum fragment length was 18, the gap length was 1000, and the reported regions are longer than 1 Kbp and have sequence identity larger than 70%.) This comparison including the alignment step takes about two minutes.

### Identification of genomic duplications

CoCoNUT also provides a chaining based strategy that is able to locate genome duplications. This is accomplished in CoCoNUT by comparing a chromosome *S *with itself. In this case, each (rare) *MEM S*[*l*_1_..*h*_1_] = *S*[*l*_2_..*h*_2_] computed in the fragment generation phase is in fact a repeat in *S *with first instance *S*[*l*_1_..*h*_1_] and second instance *S*[*l*_2_..*h*_2_]. For the special task of finding repeats, we use the algorithm from [40] for computing either maximal repeated pairs or supermaximal repeats. (A supermaximal repeat is a repeated pair such that repeated sequence does not occur as a substring of any other repeated pair.)

For detecting genome duplications, we use – as default – supermaximal repeats as fragments. In fact, supermaximal repeats can be regarded as *rare maximal repeated pairs *in which the rareness value is 4 (the size of the DNA alphabet). This follows from [[40], Lemma 3.3]. That is, a repeated pair (*S*[*l*_1_..*h*_1_], *S*[*l*_2_..*h*_2_]) is excluded if *S*[*l*_1_..*h*_1_] occurs more than 4 times in the sequence. The use of supermaximal repeats has the advantage of skipping other abundant repeats not belonging to genome duplications, and speeding up the determination of genome structures.

**Figure 8:**
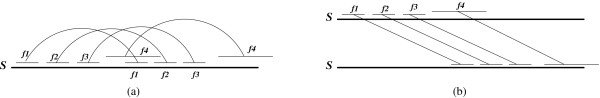
**Fragments and chaining for repeat analysis**. (a) Four repeats in sequence *S*. (b) The repeats occur as *MEMs *in a self-comparison of sequence *S*. Fragment *f*_4 _cannot be appended to the chain *f*_1_, *f*_2_, *f*_3 _because the first instance of *f*_4 _overlaps with the second instance of *f*_1_.

To identify large segmental duplications, these repeats are chained with the basic local chaining algorithm mentioned in the implementation section, but with one extra constraint: Every chain *C *= *f*_1_, *f*_2_,..., *f*_ℓ _of fragments must satisfy *end*(*f*_ℓ_).*x*_1 _<*beg*(*f*_1_).*x*_2_. That is, the first instance of the last element of a chain must not overlap with the second instance of the first element of a repeat; see Figure 8. This constraint is necessary for detecting non-overlapping repeats.

### Analyzing chromosome I of A. thaliana

**Figure 9:**
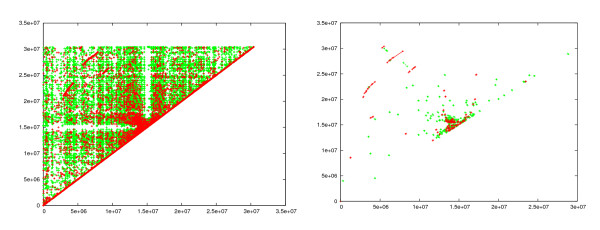
**Genome duplications in the A. thaliana chromosome I**. Left: The result after the first chaining step is applied to the *A. thaliana*. (The identity of each chain is at least 70%.) Right: The result after the second chaining step.

Locating the genome duplications within chromosome I of *A. thaliana *(accession number NC_003070) is a difficult task due to the abundance of repeated segments of different types (dispersed and tandem), and rearrangements of the repeated segments. We overcame these obstacles by a double-chaining strategy. Fragments of the type supermaximal repeats (minimum length 17, default value) were generated. These were chained with maximum gap length 250 bp. Chains spanning less than 34 bp were filtered out. Figure 9 (left) shows the resulting chains. Note that the red points near the diagonal line correspond to tandem repeats, which are abundant in this genome. Some traces of large segmental duplications can be seen in this plot. To automatically identify these, we performed a second chaining step with a gap length of 70 Kbp. Chains of length smaller than 50 Kbp were filtered out. The result, shown in Figure 9 (right), clearly reveals the genomic duplications. It is worth mentioning that our result is consistent with previous work: [41,42] found the same large segmental duplications in the *A. thaliana *genome, albeit with methods that are not fully automatic. For this comparison, CoCoNUT took less than two minutes, including the computation of the alignments (1015 MHz CPU with 1GB RAM).

### cDNA mapping

An important step in gene annotation of eukaryotes is the mapping of cDNA/ESTs to the genome. Complementary DNA (cDNA) is obtained from mRNA through reverse transcription. The cDNA is a concatenation of the exons of the expressed gene, because the introns have been spliced out. While the exons are short segments ranging from tens to hundreds of base pairs, introns can span segments of many Kbp. Expressed sequence tags (ESTs) are segments of the cDNA usually obtained by sequencing their 3' and 5' ends.

**Figure 10:**
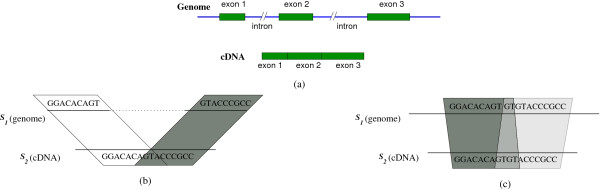
**cDNA mapping**. (a) A cDNA mapped to a genomic sequence. The exons are separated by long introns in the genome. (b) Fragments (represented by parallelograms) overlap in the cDNA sequence only. (c) The overlap is in the cDNA and in the genome.

The problem of cDNA/EST mapping is to find the gene and its exon/intron structure on the genome from which the cDNA originated; see Figure 10(a).

CoCoNUT can compare one genome to a complete cDNA or EST database. It also provides functionality for post-processing the mapped cDNA sequences (or ESTs). It clusters and reports the mapped cDNA sequences whose positions are overlapping in the genome. This feature helps in detecting alternatively spliced genes. Furthermore, CoCoNUT reports genes which are repeated in the genome.

For cDNA mapping, CoCoNUT uses a variation of the chaining method which tolerates overlaps between the successive fragments of a chain. Two fragments *overlap *if their segments overlap in one of the genomes. The rationale of allowing overlaps is twofold: First, overlapping fragments were found to be very common in cDNA mapping [3,43], and they usually occur at the exon boundaries in the cDNA; see Figure 10(b). Second, the amount of sequence covered by the chain will increase, which is crucial for both improving the sensitivity/specificity and for speeding-up the mapping. In [4,8], it was shown how to optimally solve the chaining problem with overlaps in subquadratic time based solely on range maximum queries.

After computing the optimal chain of fragments, CoCoNUT computes the alignment on the nucleotide level. The alignment step is different from standard sequence alignment because of the intron-exon structure and the splice site signals at the exon boundaries. The user can either use canonical models of the splice sites or specify a *position weight matrix *(PWM) [44].

Experimental results, presented in [4], show that our chaining algorithms achieve better specificity with the same level of sensitivity, compared to other software tools based on a seed-and-extend strategy (notably BLAT). Moreover, the algorithms work for unmasked sequences. (Using unmasked sequences results in better sensitivity than using masked sequences.) Other software tools based on the seed-and-extend strategy cannot efficiently handle unmasked sequences. To avoid redundancy, we refer the reader to [4] for more details.

## Conclusion

We have presented the software tool CoCoNUT that does not only provide functionality for whole genome comparisons (finding regions of high similarity and aligning them) of finished genomes. It is also able to detect large scale duplications, to map a cDNA/EST database to a genome, and to compare draft genomes. In principle, the latter fact allows for processing the unordered sets of sequences (reads, contigs) delivered by new sequencing technologies (like 454 [45], Solexa [46], or SOLID [47]). However, we have not yet evaluated such an application. There are other software tools which solve one of the mentioned tasks individually but to the best of our knowledge CoCoNUT is the first software tool with such a broad spectrum of applications. This feature makes it especially attractive to users who have to solve a wide range of comparative genomics problems.

CoCoNUT uses several new algorithms developed by the authors of this article, notably an algorithm for the space efficient computation of rare *multiMEMs *[5] based on enhanced suffix arrays [40] and new chaining algorithms [7,8]. As a consequence, CoCoNUT is fast and memory efficient, and users will certainly appreciate that it scales well for large input sizes.

## Availability and requirements

CoCoNUT is freely available for non-commercial users. For details and tool download, see http://toolcoconut.org. Mirror site: http://www.nubios.nileu.edu.eg/tools/CoCoNUT.

***Project name***: **C**omputational **C**omparative ge**N**omics **U**tility **T**oolkit (CoCoNUT)

***Project home page***: http://toolcoconut.org; Mirror site: http://www.nubios.nileu.edu.eg/tools/CoCoNUT

***Operating system(s)***: Unix/Linux (windows version under development)

***Programming language***: C, C++, Perl

***License***: free for non-commercial purposes

***Any restrictions to use by non-academics***: see license agreement on the tool home page

## Authors' contributions

EO initiated and lead the project to develop a versatile software tool for comparative genomics. The roots of the project go back to a time when all authors worked jointly at Bielefeld University. All authors contributed to theoretical developments which form the basis of CoCoNUT. MA developed and tested the software, except for the programs ramaco and multimat which were implemented by SK. All authors wrote and approved the manuscript.

## Supplementary Material

Additional file 1 **The coordinates of the regions of high similarity and the synteny blocks.** Additional file 1 contains the coordinates of the regions of high similarity and synteny blocks (Tables 1 and 2 respectively) of the (unmasked) X-chromosomes of human, mouse, and rat. Click here for file
