# Supplementary material for: CoCoNUT: an efficient system for the comparison and analysis of genomes
Source: BMC Bioinformatics. 2008 Nov 12;9:476. doi: 10.1186/1471-2105-9-476 (PMC3224568; doi:10.1186/1471-2105-9-476)
Supplement: Additional file 1 — The coordinates of the regions of high similarity and the synteny blocks. Additional file 1 contains the coordinates of the regions of high similarity and synteny blocks (Tables 1 and 2 respectively) of the (unmasked) X-chromosomes of human, mouse, and rat. [file 1471-2105-9-476-S1.pdf]

Additional File 1

The coordinates of the regions of high similarity and the synteny blocks

Tables 1 and 2 contain the coordinates of syntenic regions and synteny blocks of the (unmasked) X-chromosomes of human, mouse, and rat , respectively.

Table 1: Syntenic regions of the (unmasked) X-chromosomes of human, mouse, and rat.

	Human		Mouse		Rat	
Strands	L_h	start, end	L_m	start, end	L_r	start , end
+++	467886	9179897,9647783	306667	74601660,74908327	330306	63062225,63392531
+-+	7664681	10397756,18062437	8577044	157846009,166423053	9378792	44776455,54155247
+-+	4432675	18170131,22602806	4683907	153009563,157693470	5148650	54799697,59948347
+-+	763034	22951211,23714245	914924	151647683,152562607	1105377	60532870,61638247
+--	2384569	23761438,26146007	2337120	89325266,91662386	2705871	79183634,81889505
+--	1729557	26365545,28095102	2166835	86056279,88223114	2487414	76332109,78819523
+--	5351854	28282039,33633893	6110642	79590728,85701370	6962713	68802619,75765332
++-	4702556	37778101,42480657	5151951	9462801,14614752	5382332	19651677,25034009
++-	3965312	43386232,47351544	4304047	16182664,20486711	5240844	12512239,17753083
+-+	1986585	48205065,50191650	1408850	6445357,7854207	1510003	26247496,27757499
+--	900113	50356432,51256545	586420	5624416,6210836	651933	28682370,29334303
+--	1962455	53094295,55056750	1802581	147001098,148803679	2062863	39806481,41869344
++-	1012414	55760911,56773325	584556	149574495,150159051	658014	37726641,38384655
+++	875376	62486692,63362068	446035	92220802,92666837	450520	82564598,83015118
+++	1526461	64055659,65582120	1077704	92837515,93915219	1070298	83221410,84291708
+++	6888334	65933885,72822219	5887123	94702799,100589922	6143838	85220128,91363966
+++	1304692	73354932,74659624	1097929	100768249,101866178	1136879	91563406,92700285
+++	4274545	75311154,79585699	2789920	102276737,105066657	2965532	93154013,96119545
+++	605608	79813700,80419308	412001	105933422,106345423	538581	97004802,97543383
+++	2821142	80908133,83729275	2082860	106822494,108905354	2235463	98061015,100296478
+++	2652542	84114886,86767428	2302683	109354090,111656773	2462592	100834259,103296851
+++	320868	89217373,89538241	410663	115709122,116119785	409959	107912648,108322607
+++	1620026	90141371,91761397	1300361	116720470,118020831	1524141	109034353,110558494
+++	479329	92656677,93136006	661620	119248969,119910589	607508	112014099,112621607
+++	6548268	94525627,101073895	6926806	124617187,131543993	7937683	114547610,122485293
+++	3723370	103468047,107191417	3693930	133760226,137454156	4184395	124578908,128763303
++-	5453627	107294914,112748541	5054520	137609562,142664082	5253171	32226081,37479252
++-	824132	113550984,114375116	763542	143225317,143988859	1054149	30376153,31430302
++-	1495766	115972091,117467857	1524204	21858778,23382982	1830904	9720301,11551205
++-	1356359	117513501,118869860	1220719	33428526,34649245	1234991	7802748,9037739
++-	544348	119271294,119815642	625355	35545389,36170744	644718	6523738,7168456
++-	5451691	120251711,125703402	5575062	36756736,42331798	5888930	7561,5896491
+++	663947	125919584,126583531	795718	42576547,43372265	1129929	131105514,132235443
+++	3123613	127244138,130367751	3583760	43788329,47372089	3730479	132791653,136522132
+++	3325165	130570799,133895964	2916095	47823573,50739668	3263314	137091673,140354987
+++	5023878	134874203,139898081	4868963	53841699,58710662	5919941	141126905,147046846
+--	437285	139947609,140384894	446857	58735343,59182200	393801	147069212,147463013
+++	2848517	141198752,144047269	3069160	59868237,62937397	3330696	148264712,151595408
+++	796409	144562993,145359402	829667	63714684,64544351	874457	152409707,153284164
+++	1964449	146271337,148235786	2146514	65322909,67469423	2518355	154240620,156758975
+++	653752	149789168,150442920	407696	68728267,69135963	305356	157023298,157328654
+++	2040751	151108706,153149457	1698670	69690361,71389031	1703832	158405356,160109188

Syntenic regions of the (unmasked) X-chromosomes of human, mouse, and rat, obtained after the second chaining step. The first column contains the orientation of the syntenic regions. For example, "++-" means that the region is on the forward strand of the human and mouse X-chromosomes and on the reverse strand of the rat X-chromosome. The columns titled "L_h", "L_m", and "L_r" show the lengths of the regions. The column titled "start,end" contains the start and end position (separated by a comma) of the respective region.

Table 2: Syntenic blocks of the (unmasked) X-chromosomes of human, mouse, and rat.

	Human		Mouse		Rat	
`Strands`	L_h	start, end	L_m	start, end	L_r	start , end
`+++`	467886	9179897,9647783	306667	74601660,74908327	330306	63062225,63392531
`+-+`	13316489	10397756,23714245	14775370	151647683,166423053	16861792	44776455,61638247
`+--`	9872455	23761438,33633893	12071658	79590728,91662386	13086886	68802619,81889505
`++-`	9573443	37778101,47351544	11023910	9462801,20486711	12521770	12512239,25034009
`+-+`	1986585	48205065,50191650	1408850	6445357,7854207	1510003	26247496,27757499
`+--`	900113	50356432,51256545	586420	5624416,6210836	651933	28682370,29334303
`+--`	1962455	53094295,55056750	1802581	147001098,148803679	2062863	39806481,41869344
`++-`	1012414	55760911,56773325	584556	149574495,150159051	658014	37726641,38384655
`+++`	44704725	62486692,107191417	45233354	92220802,137454156	46198705	82564598,128763303
`++-`	7080202	107294914,114375116	6379297	137609562,143988859	7103099	30376153,37479252
`++-`	9731311	115972091,125703402	20473020	21858778,42331798	11543644	7561,11551205
`+++`	13978497	125919584,139898081	16134115	42576547,58710662	15941332	131105514,147046846
`+--`	437285	139947609,140384894	446857	58735343,59182200	393801	147069212,147463013
`+++`	11950705	141198752,153149457	11520794	59868237,71389031	11844476	148264712,160109188

Syntenic blocks of the (unmasked) X-chromosomes of human, mouse, and rat, obtained when using the corresponding option of CoCoNUT. The first column contains the orientation of the syntenic blocks. For example, "`++-`" means that the block is on the forward strand of the human and mouse X-chromosomes and on the reverse strand of the rat X-chromosome. The columns titled "L_h", "L_m", and "L_r" show the lengths of the blocks. The column titled "start,end" contains the start and end position (separated by a comma) of the respective region.
